# Tribological, biocompatibility, and antibiofilm properties of tungsten–germanium coating using magnetron sputtering

**DOI:** 10.1007/s10856-020-06477-4

**Published:** 2021-01-20

**Authors:** Mustafa Şükrü Kurt, Mehmet Enes Arslan, Ayşenur Yazici, İlkan Mudu, Elif Arslan

**Affiliations:** 1 Physics Department, Faculty of Science, Erzurum Technical University, Erzurum, Turkey; 2 Molecular Biology and Genetics Department, Faculty of Science, Erzurum Technical University, Erzurum, Turkey

## Abstract

In this study, borosilicate glass and 316 L stainless steel were coated with germanium (Ge) and tungsten (W) metals using the Magnetron Sputtering System. Surface structural, mechanical, and tribological properties of uncoated and coated samples were examined using SEM, X-ray diffraction (XRD), energy-dispersive spectroscopy, and tribometer. The XRD results showed that WGe_2_ chemical compound observed in (110) crystalline phase and exhibited a dense structure. According to the tribological analyses, the adhesion strength of the coated deposition on 316 L was obtained 32.8 N, and the mean coefficient of friction was around 0.3. Biocompatibility studies of coated metallic biomaterials were analyzed on fibroblast cell culture (Primary Dermal Fibroblast; Normal, Human, Adult (HDFa)) in vitro. Hoescht 33258 fluorescent staining was performed to investigate the cellular density and chromosomal abnormalities of the HDFa cell line on the borosilicate glasses coated with germanium–tungsten (W–Ge). Cell viabilities of HDFa cell line on each surface (W–Ge coated borosilicate glass, uncoated borosilicate glass, and cell culture plate surface) were analyzed by using (3-(4,5-Dimethylthiazol-2-yl)-2,5-diphenyltetrazolium bromide) cytotoxicity assay. The antibiofilm activity of W–Ge coated borosilicate glass showed a significant reduction effect on *Staphylococcus aureus* (ATCC 25923) and *Pseudomonas aeruginosa* (ATCC 27853) adherence compared to control groups. In the light of findings, tungsten and germanium, which are some of the most common industrial materials, were investigated as biocompatible and antimicrobial surface coatings and recommended as bio-implant materials for the first time.

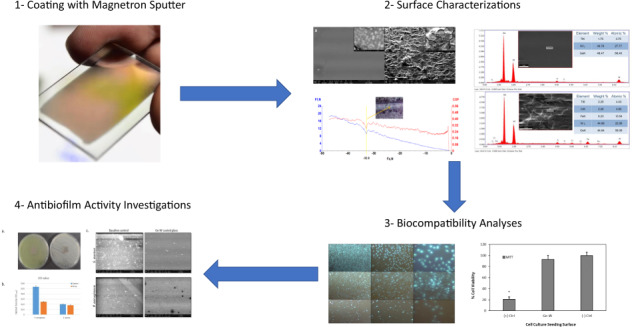

## Introduction

Biomaterials have been studied for more than 50 years in the field of medicine and engineering to fulfill the vital function of tissues in the human body with natural or synthetic material types [1]. Biomaterials can be bio-tolerant, bioinert, bioactive, and biodegradable materials that enhance a biological function in the body. These properties, which biomaterials must possess, enable the material to be integrated with the tissue in the body and ensure long-life assistance. Biocompatibility, bioactivity, osteointegration, mechanical, and corrosion resistance properties have been investigated to find desired conditions and properties that can be used in the human body for complementing a function. Artificially developed biomaterials in materials science are diversified into ceramics, composites, polymers, and metals [2–4]. Implant materials used as biomaterials should not cause any inflammation and toxicity in the body. The biomaterials that are inserted into the body to function successfully without causing any undesired response is called biocompatibility [5]. One of the most important factors affecting biocompatibility is the reaction of the body to the material, and the other is the chemical structure of the material. The appropriate reaction expected to be given to the material by the immune system is that the substance does not cause inflammation on the material surface and exhibits integration with the body [6]. In addition to the integration of the material into the body, electrochemical and mechanical phenomena occur on the surface of the material and the mixing of ions released from the material into the body’s liquid, blood, or tissue occurs. For example, Zn^+^ ions from biomaterials contribute to protein formation because zinc is present in the chemical content of many enzymes and amino acids. It is known that heavy metals such as arsenic and cadmium cause zinc to separate from zinc-bound body structures, thereby disrupting the function of certain organs in the body and even causing cancer [7]. In this case, the toxic effect or bioactive properties of the material should be taken into account. Bacterial biofilm formation on biomaterials is another important problem for development implants. Bacterial contamination on implant causes infection to lead to difficult treatment. Antibiotic coating of implants against infections is an alternative method. However, these coatings are not preferred because of increasing antibiotic resistance [8]. Therefore, as well as the implant surfaces being biocompatible, they should also have antibiofilm properties. To this end, this is the first study on W–Ge coated materials and investigation their tribological, biocompatibility, antibiofilm properties.

Germanium (Ge) is a metalloid, which is known chemically similar to silicon (Si), and mostly used in the electronics industry. This material potentially useful for not only optics, sensors, and catalysis, but also can be used as antimicrobial agents. The potential antimicrobial properties of Ge compounds were proved against the human pathogenic bacteria [9]. Also, the antimicrobial properties of elemental Ge have been shown in suspension against both *Staphylococcus aureus* and *Pseudomonas* *aeruginosa* planktonic [10]. Ge is a dietary trace element that is needed for some biological functions such as lowering blood pressure and maintaining the normal range of glucose, pH, sodium, and potassium [11]. Ge naturally exists in food at very low concentrations and it is taken by people in the ranges of 0.4–3.4 mg daily [12–15]. Nagata et al. [13] reported that fatal cases of acute renal failure in humans were caused by high dosage (600 mg daily) and long-term (18 months) GeO_2_ intake. Lück et al. [14] also reported that renal failure was observed in a patient who was taken Ge-132 (including 76 g of elemental Ge) over 6 months. However, on potential, cytotoxic effects of Ge on the human body in MgGe alloys were investigated by Bian et al. [11]. This study showed that low concentration of Ge (below ~3 wt%) in MgGe alloys possess great histocompatibility, cytocompatibility, corrosion resistance, and mechanical properties. The combination of such properties makes these alloys great candidates for potential orthopedic implant materials. Furthermore, the study on the therapeutic effect of Ge-132 showed that Ge causes increased cell activity of osteoblast, including alkaline phosphate metabolism, and prevents mineral decomposition in osteoporosis [16]. Moreover, Ge-132 prevented osteoporosis patients from bone mass reduction caused by parathyroid hormone [17]. Thus, the studies on Ge show that high concentrations of GeO_2_ or inorganic compounds of Ge are relatively toxic, however, low concentrations of Ge contribute to the physiological functions in the human body. To avoid adverse effects of Ge-132, Ge, and GeO_2_, the lowest ranges observed are between 42 and 1380 mg/d for average weight (60 kg) adult [11].

Extraordinary physical and mechanical properties of tungsten, especially good wear resistance, high melting point, and hardness made it attractive for using in many areas such as coatings on heat pipes, corrosion-resistant coatings, nuclear power systems, and microelectronic systems [18–21]. Previous studies showed that many properties of tungsten have been investigated, and all these properties are related to their tribological behavior. On the other hand, such behavior of tungsten, especially how elevated temperatures affect its performance, and the relationship between microstructure processing and property have not been understood well enough. Therefore, it is worth investigating the tribological properties of tungsten to improve effective application materials. Estevea et al. reported that tungsten carbide (WC) was deposited on steel substrates with depositing tungsten (W) intermediate layer using r.f. magnetron sputtering system. The results showed that WC overlayer contributed to enhancing adhesion properties and the W intermediate layer provided a high degree of reduction of wear rate [22]. Tribological properties of tungsten and tungsten mechanical or chemical alloys coating have been completed from room to high temperatures by researchers in many studies [23–26]. These researches demonstrate that increasing the substrate temperature causes changing the microstructures of tungsten, and these changes strongly affected the tribological properties of tungsten coatings. Only the study came across in the literature on W and Ge sputtering together carried out by Piedade et al. [27]. In this study, W–Ge–N was deposited using reactive RF magnetron sputtering. The density of composition was enhanced by adding both germanium (Ge) and nitrogen (N). Increasing N content in composition contributed to the hardness of the alloy while increasing Ge content in composition leads to a decrease in the hardness value.

In order to overcome the aforementioned issues such as toxicity, inflammation, bacterial contamination, and reaction of the body, some effective posttreatment of the biomaterials is suggested by coating them biocompatible and corrosion-resistant materials [28]. The most common methods to obtain a desired surface modification of biomaterials are coating them via the use of physical and chemical vapor depositions. Thus, surface modification of biomaterials allows enhancing their properties as required, such as biocompatibility, antimicrobial activity, and corrosion resistance with maintaining their genuine properties [29–32].

In this study, the W–Ge alloy was investigated as a composition material due to the potential biologic properties of Ge and extraordinary physical and mechanical properties of tungsten as mentioned above. Ti was deposited as the first layer on substrates before coating W–Ge due to obtaining good adhesion properties to improve the interfacial strength [33]. This is the first study on W–Ge composition as an implant material, which encloses favorable tribological, biocompatibility, antibiofilm properties.

## Materials and methods

### Experimental details

Before the magnetron sputtering process, borosilicate glass (for biological tests) and 316 L stainless steel (for physical tests) substrates were cleaned with isopropanol and distilled water, respectively, using the ultrasonic cleaner for 15 min for each, and cleaning progress ended with air blow-drying. Two inches diameter titanium (Ti), germanium (Ge), and tungsten (W) solid targets with purity (99.99%) were used to prepare samples. Before deposition, to achieve better surface activity and to remove contaminants on the target surface, each target was cleaned using a plasma cleaning process for 10 min. All samples were prepared using a magnetron sputtering system (Nanovak, Model: NVTS-400, 2TH2SP) equipped with two RF, one DC sputter, and two thermal sources. All the depositions were carried out at a base pressure of ∼4 × 10^−6^ mTorr and a working pressure of ∼2 × 10^−3^ mTorr in the argon (Ar) atmosphere. Initially, Ti buffer layer was deposited on the substrate. Afterward, Ge and W were deposited on Ti buffer layer using the co-deposition technique with deposition rates of 50% Ge and 50% W. Deposition rates of samples were determined using a quartz thickness monitor. The deposition temperature during the sample preparation was around room temperature (25–35 C°) and the distance from the target to the substrate was 10 cm. The power, 115 W (RF), 115 W (RF), and 300 V (DC), was applied to Ti, Ge, and W targets, respectively. 200 nm Ti and 1000 nm Ge–W were deposited on the substrates. Physical characterization of all samples was carried out using X-ray diffraction (XRD) (EXPLORER-GNR), scanning electron microscope (SEM) (Quanta FEG 250), energy-dispersive spectroscopy (EDS) (Edax), and tribometer (Bruker UMT).

### Static corrosion investigation

Commercially available Dulbecco’s modified Eagle’s medium (DMEM, high glucose 4.5 g/L, +glutamine, w/o pyruvate, Sigma^®^, USA) included 10% fetal bovine serum (FBS, Sigma^®^, USA) was used to analyze static corrosion behavior of W/Ge coated the borosilicate glass surface. Coated samples were placed in a six-well plastic culture plate with 2 ml DMEM immersion. Culture plates were incubated for 7 days at 37 °C under a humidified atmosphere of 95% air and 5% CO_2_. After static corrosion was completed, samples were removed from the media and washed with distilled water for scanning electron microscope (SEM) investigations. Surface topology and material components were observed via SEM and EDS analyses [34].

### Cell culture and seeding

Human fibroblast (HDFa) cell culture (ATCC^®^ PCS201 012^™^) was grown in T25 cell culture flask by using DMEM (high glucose 4.5 g/L, +glutamine, w/o pyruvate, Sigma^®^, USA) included 10% FBS (Sigma^®^, USA) and 1% Penicillin/streptomycin (10,000 IU/ml 10,000 µg/ml, Thermo Fisher Scientific^®^, USA) at 37 °C, 5% CO_2_ humidified incubator. After the culture reached the confluency, cells were detached from the flask surface via the use of Trypsin-EDTA (0.25%, Sigma^®^, USA) solution. The cell culture was seeded as 1 × 10^5^ number of cells on the tungsten–germanium coated borosilicate glasses in 12-well plate and incubated for 24 h at 37 °C, 5% CO_2_ incubator to analyze attached cells. Experimental groups were classified as coated materials, uncoated borosilicate glasses, and negative control (cell culture plate surface), and each group was analyzed in triplicate samples.

### Fluorescence staining

Hoescht 33258 staining was performed to detect the cellular density and chromosomal abnormalities of the HDFa cell line on the borosilicate glasses coated with tungsten–germanium. After 24 h of incubation, cell cultures in 12-well plates were washed with phosphate-buffered saline (PBS, 1X, pH 7.4, Thermo Fisher Scientific^®^, USA). Coated and uncoated materials were placed in a fresh culture plate with the aid of sterile tweezers. The cell cultures were fixed by using 4% glutaraldehyde solution at +4 °C for 30 min. Fixed cells were washed with 1x PBS and 1 µM Hoescht 33258 fluorescent dye added to each well and incubated for 5 min at room temperature. The stained sample surface was monitored with a fluorescent microscope at ×10, ×20, and ×40 magnifications.

### Cell viability analysis

3-(4,5-Dimethylthiazol-2-yl)-2,5-diphenyltetrazolium bromide (MTT, Cayman Chemical Company^®^, Ann Arbor, MI, USA) solution was used according to the manufacturer’s instructions. Briefly, coated and uncoated materials were rinsed with DMEM media and 0.5 ml of fresh DMEM + 10% MTT solution was applied to the samples and incubated for 3 h at 37 °C, 5% CO_2_ humidified incubator. Formazan crystals were dissolved by using dimethyl sulfoxide (Sigma-Aldrich^®^), and each well was analyzed using a plate reader (Epoch™, USA) at 570 nm wavelength.

### Bacterial culture preparation

*S. aureus* (ATCC 25923) and *P. aeruginosa* (ATCC 27853) were kindly provided by Dr. İskender Karaltı (Department of Medicine, University of Yeditepe, İstanbul, Turkey). These bacteria were cultured in Mueller Hilton broth (MHB, Oxoid) medium at 37 °C overnight. The bacterial suspension was adjusted to 0.5 McFarland standard in MHB to evaluate antimicrobial and antibiofilm properties.

### Halo inhibition zone tests

Antimicrobial activity was performed using halo inhibition zone tests [35]. Briefly, all samples were sterilized using 70% ethanol for overnight and followed by washing sterile distilled water. Then, this test was performed using *S. aureus* and *P. aeruginosa*. Overnight cultures were adjusted to 0.5 McFarland and seeded on the MHA plate. Inhibition zones were checked.

### Evaluation of antibiofilm activity by SEM

Borosilicate glasses coated with tungsten–germanium were sterilized for 24 h with UV after 10 min 70% EtOH incubation. Borosilicate glasses coated with tungsten–germanium were placed in a 12-well plate and then 0.5 McFarland bacterial cells were added. After incubation for 48 h at 37 °C, they were washed with a 0.25% Trypsin solution to collect the biofilm from the sample. The solution was evaluated by the 600 nm the OD measurement. W–Ge coated borosilicate glass samples were sterilized in 70% Ethanol at 30 min and UV at 48 h. Then, samples and bacterial cultures seed in six-well plate in MHB medium. Borosilicate glasses are used for baseline control. Incubated samples were washed twice with PBS and fixed 2.5% glutaraldehyde at room temperature for 30 min. After fixation, samples dehydrated in ethanol series (20, 50, 80, 90, 99.9%) for 10 min. Finally, samples were observed via scanning electron microscope (SEM-Quanta FEG 250) [31].

### Statistical analysis

Statistical analyses of the numerical data obtained from the studies were performed with GraphPad Prism 7; ANOVA Dunnett’s multiple comparison test was used following the two-way ANOVA analysis for statistical evaluation and the statistical significance level was accepted as *p* < 0.05.

## Results and discussion

Materials used in biomedical applications were applied in different forms and used for specific properties to accomplish a particular task. Materials such as metals, which were shown to enhance certain functions in the human or animal body, were often used as implants [36]. In previous studies, biomaterials were expected to exhibit biomechanical properties comparable to living tissues without side effects. The main characteristics that determine the suitability of a material for biomedical implant applications could be listed as biocompatibility, corrosion resistance, and bio-function [37]. In this study, W–Ge alloy coating on the borosilicate glass was performed via the magnetron sputtering technique. Surface characteristics were investigated by using SEM, EDS, XRD, scratch, and static corrosion assays. Biocompatibility was analyzed via using HDFa cell line, and cytotoxicity of the coating was studied by MTT cell viability assay. Finally, the antimicrobial property of the W–Ge coating was inspected by antibiofilm assay by using *S. aureus* (ATCC 25923) and *P. aeruginosa* (ATCC 27853) bacteria strains.

**Fig. 1:**
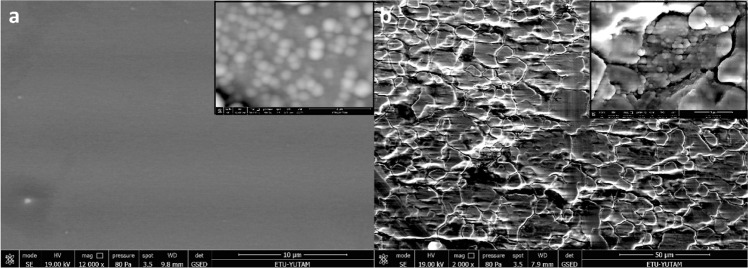
Scanning electron microscope (SEM) images of W–Ge coated: **a** boron silicate glass 10 and 2 µm (inset picture); **b** 316 L stainless steel 50 and 5 µm (inset picture)

Figure 1 displays SEM images of 316 L stainless steel and borosilicate glass surfaces of Ti and W–Ge coating samples. As can be seen in Fig. 1b, after coating 316 L stainless steel morphology still has the typical 316 L microstructure [38]. Figure 1a shows the morphology of coated boron silicate glass. As known boron silicate glass is an amorphous material, due to this the atomic arrangement of the material is densely packed [39]. Unlike 316 L microstructure, this leads to quite a smooth and uniform surface profile without any grain boundaries at the substrate surface. Since the surfaces of the coated samples have no deformation such as cracks and pores.

**Fig. 2:**
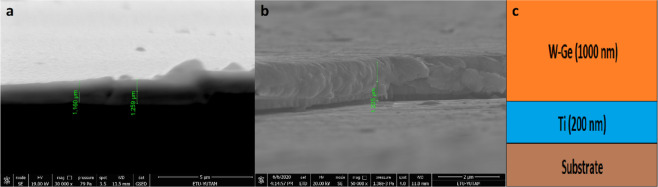
SEM cross-section of coating materials on **a** borosilicate glass, **b** 316 L stainless steel, and **c** schematic demonstration of W–Ge and Ti coating layers on substrates

As shown, Fig. 2 coating samples consist of two layers on borosilicate and 316 L stainless steel substrate, wherein the first layer is 200 nm Ti and the second layer is 1000 nm W–Ge composition, respectively. It can be seen from the SEM cross-section image of Fig. 2a, b that mean thickness of deposition materials is around 1200 nm (200 nm Ti and 1000 nm W–Ge) as measured during the deposition using a crystal thickness monitor. This rate of deposition thickness is reasonably sufficient for wear-resistant coating applications [40]. Investigation of the cross-section SEM image of the W–Ge coating on borosilicate glass revealed that the coating structures are smooth, dense, and poreless.

**Fig. 3:**
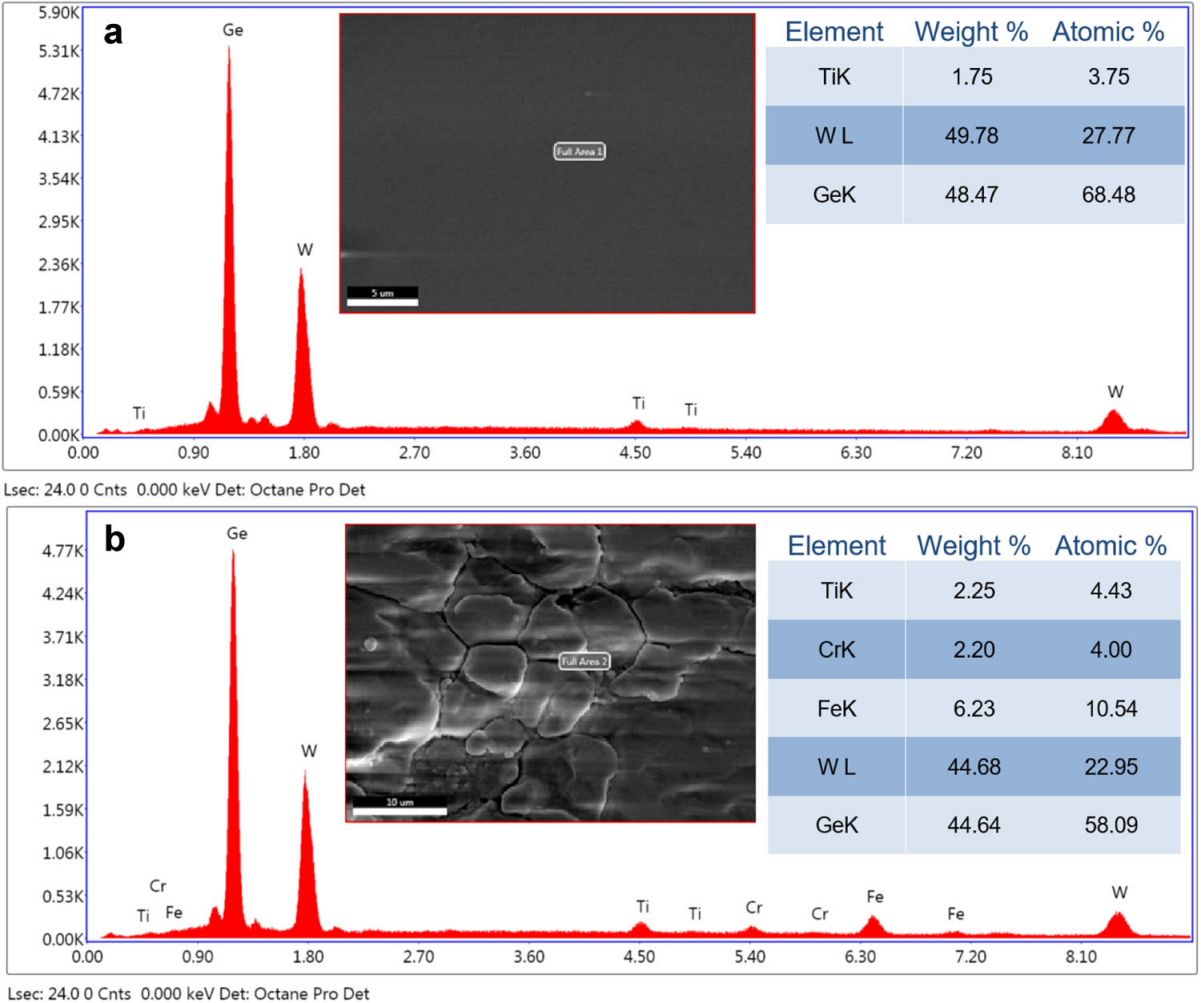
EDS images of W–Ge coated samples on: **a** borosilicate substrate; **b** 316 L stainless steel substrate

Figure 3 shows that the results of EDS were performed on both borosilicate and 316 L stainless steel substrates. Figure 3a shows the atomic contents on the borosilicate surface. The percentages of atomic contents are 4.01% Ti came from the buffer layer, 27.32% W and 68.67% Ge came from the composition layer. Also, Fig. 3b shows the atomic contents of coating deposition on 316 L stainless steel substrate. The percentages of atomic contents are 4.43% Ti and 4.0% Cr, 10.54% Fe came from the buffer layer and substrate, respectively, whereas 22.95% W and 58.09% Ge came from the composition layer. Furthermore, the elemental weight of W and Ge in the composition is almost the same (50–50%) as measured using a crystal thickness monitor. During the deposition process, element contents of compositions are mainly affected by deposition parameters such as sputtering power and gas flow rates when using magnetron sputter technology [41]. In order to obtain the same elemental weight for composition materials (W–Ge), W and Ge were deposited using DC sputter (300 V) and RF sputter (115 W), respectively, in 5 mTorr argon (Ar) environment.

**Fig. 4:**
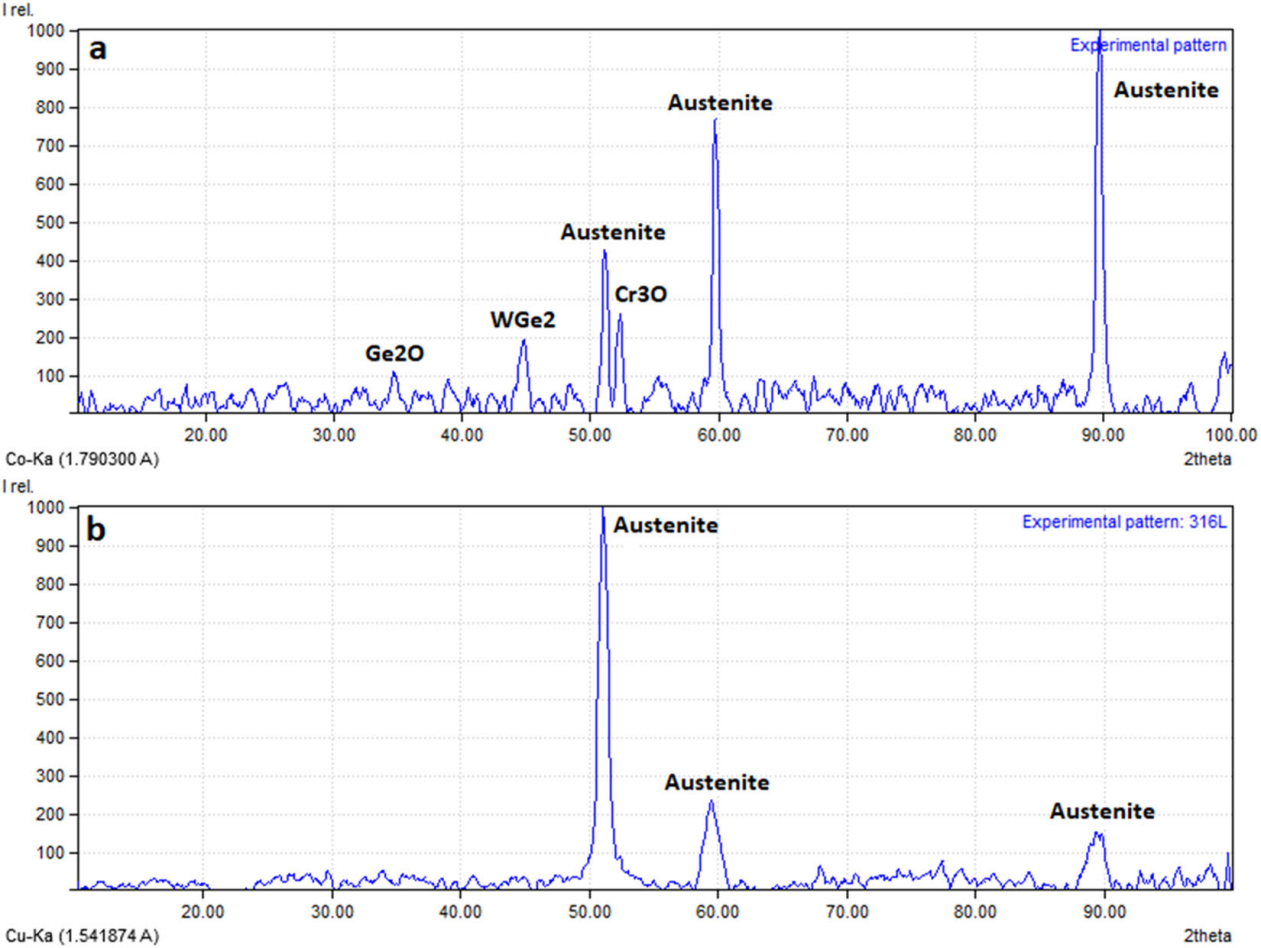
XRD patterns of **a** W–Ge coating sample; **b** uncoated 316 L stainless steel

Figure 4b shows the XRD spectrum of the uncoated 316 L stainless steel substrate for comparison purposes. It can be seen from Fig. 4b that typical XRD patterns were observed for uncoated 316 L with 2θ Fe peak values of 51°, 59°, and 89° as expected [42]. Figure 4a shows the XRD spectrum of the coated 316 L stainless steel substrate with 200 nm Ti buffer layer and 1000 nm W–Ge composition layer, respectively. As can be seen from Fig. 4a, the small thickness or porous nature of the coating causes austenite diffusion from the substrate to the surface layer. 2θ peak value of 52° shows the Cr_3_O (JCPDS: 96-152-8030), which was coming from a passive oxide layer on the 316 L substrate. As mentioned above and showed in Fig. 3, the deposition rate was arranged to be 50% W and 50% Ge for deposition. Reactive sputtering of W and Ge leads to the chemical composition of WGe_2_, which is shown in Fig. 4a with 2θ (110) peak value of 44.86°, is matched with the JCPDS value of 00-071-1271. Also seen in Fig. 3, the atomic constitution of Ge is more than twice when compared to the W. Since the Ge atoms, which were not chemically compounded with W, were oxidized, most probably by the oxide layer on the 316 L substrate. The small peak shown with 2θ peak value of 34° in Fig. 4a corresponds to the oxidized Ge atoms (JCPDS: 96-900-6858). The results of the XRD analysis show that the coating has crystal structures. The crystalline grains are clearly seen in Fig. 1a inset picture. According to the XRD results, (110) orientation of WGe_2_ is the dominant peak in W–Ge coating.

**Fig. 5:**
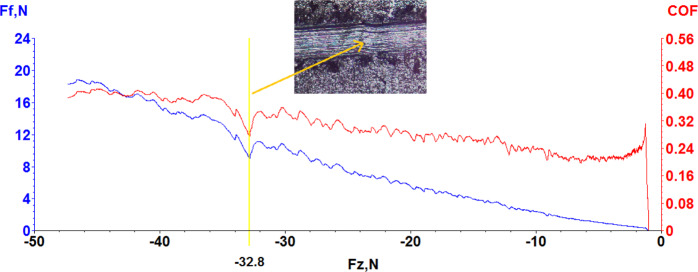
The critical load value and friction coefficients of W–Ge coating sample against to normal load, and adhesive failure seen inset picture

Scratch test is a well-known technique when determining the adhesion properties of materials [43]. The conventional scratch test was carried out to the W–Ge coating to investigate critical adhesion load (Lc) value at room temperature under atmospheric conditions. A tribometer (Bruker UMT) has been employed to investigate the adhesion behavior at the interface of the coating deposition and the substrate, revealed with critical loads (Lc). The scratch test was carried out to backward direction under the experiment parameters with 250 N/min load rate, 50 N maximum load, and 10 mm scratch length. Figure 5 shows the values of friction coefficient (COF) and friction force (Ff) against normal force (Fz) and also critical load (*L*_*c*_), which were obtained from the scratch test. Initially, the friction coefficient has the values around to 0.24, and slightly increased to around 0.32. Because of the smooth morphology of the sample surface, the friction coefficient was found quite stable, and the mean friction coefficient was investigated to be around the value of 0.3. With increasing normal force from 0 to 50 N, as expected Ff also increased to around 20 N. It is known that, in order to investigate the critical adhesion load value, normal force increased gradually and when the scratch tip reached to the substrate, which causes to the adhesive failures in the coating sample and this leads to dramatic changes on the friction coefficient and Ff at the data. From this point of view, the critical load value of the coating deposited on 316 L substrate obtained from the data is 32.8 N. Also, adhesive failure occurred as a result of the radial crack in the sample is shown inset picture in Fig. 5. According to the literature, after removing samples from the ultra-high vacuum condition to the atmospheric environment, oxidation of the metals can be observed at the film surface [44]. XRD analyses show that with WGe_2_ composition, Ge_2_O and Cr_3_O metallic oxides were also seen in the coating deposition due to either the oxide layer on the substrate or oxidization after removing the deposition chamber. As a result of these oxidations of metals and crystallized form of WGe_2_ in the film, wear properties and the friction coefficient of the film became lower. In the literature, friction coefficient and hardness values were shown inversely proportional to each other according to Archard’s model [45]. The low friction coefficient value indicates that high hardness occurs at the coating deposition as expected due to the high hardness nature of the deposition materials. The mean friction coefficient value obtained from the scratch test is adequately low (~0.3), when compared with uncoated 316 L stainless steel values (above ~0.5) [46–48]. General results show that the friction coefficient value of the crystalline W–Ge coating sample had a lower value than the uncoated 316 L stainless steel substrate because of high density and hardness in coating structures.

**Fig. 6:**
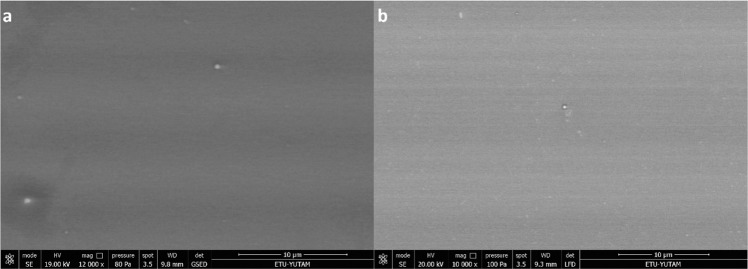
Scanning electron microscope (SEM, FEI inspect S50 SEM) image W–Ge coated borosilicate surface. **a** W–Ge coating before static corrosion test; **b** static corrosion of W–Ge under DMEM immersion for 7 days

**Fig. 7:**
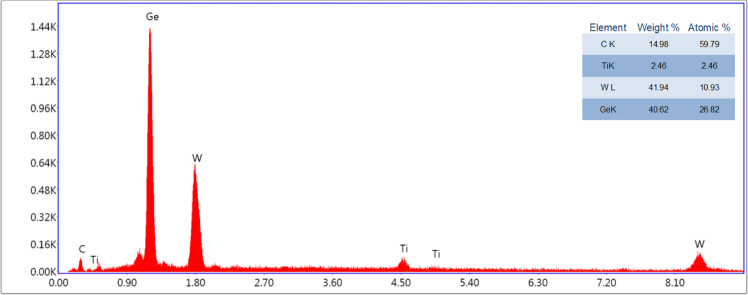
Energy-dispersive X-ray spectroscopy (EDS, EDX) results of W–Ge coated borosilicate glass surface that show the atomic ratios of W/Ge (at.%) after 7 days of static corrosion under DMEM immersion

To investigate the corrosion behavior of W–Ge coating, static corrosion analysis was performed by using DMEM in place of the SBF (simulated body fluid) [34]. After 7 days of incubation with DMEM, there was no observable change in surface morphology under an electron microscope (Fig. 6) and also EDS analysis puts forth that there was no significant change in the atomic ratio of W/Ge molecules. On the other hand, carbon peaks were seen in all EDS results of corrosion analysis, and these peaks were coming from the unwashed DMEM media, which was used for corrosion tests (Fig. 7).

**Fig. 8:**
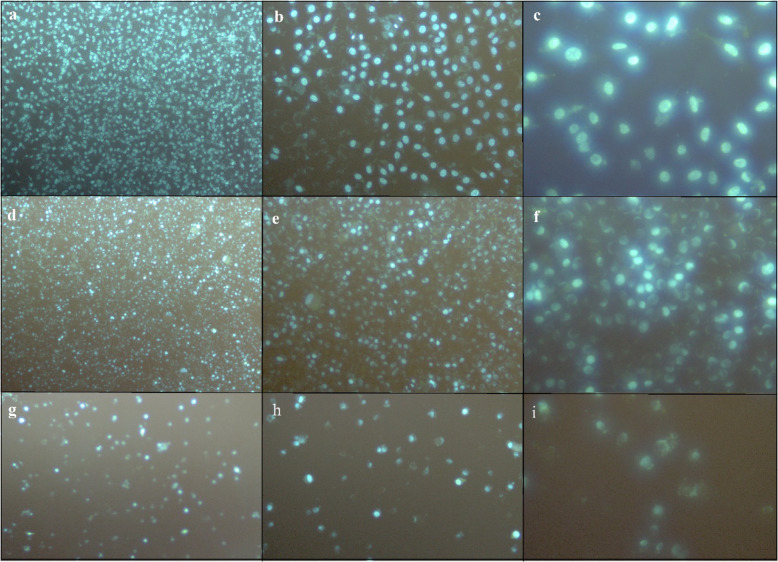
Immunofluorescence microscopy images of human fibroblast (HDFa) cell culture attachments on different surfaces after 24 h. **a** W–Ge coated borosilicate surface (×10 magnification), **b** W–Ge coated borosilicate surface (×20 magnification), **c** W–Ge coated borosilicate surface (×40 magnification), **d** cell culture plate bottom surface (×10 magnification), **e** cell culture plate bottom surface (×20 magnification), **f** cell culture plate bottom surface (×40 magnification), **g** uncoated borosilicate surface (×10 magnification), **h** uncoated borosilicate surface (×20 magnification), **i** uncoated borosilicate surface (×40 magnification)

**Fig. 9:**
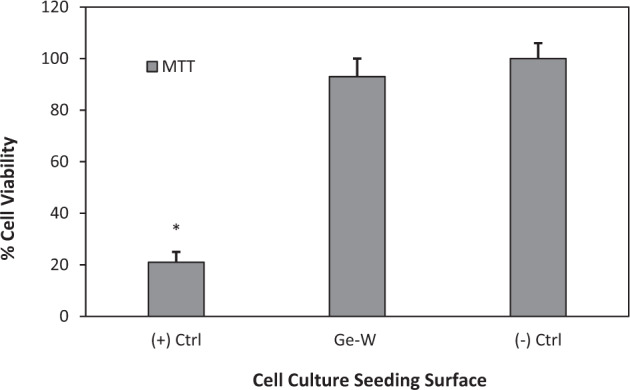
MTT cell viability analysis of uncoated borosilicate surface ((+) Ctrl), W–Ge coated borosilicate surface and cell culture plate bottom surface ((−) Ctrl). Symbol (*) represents significant decrease in cell viability compared to (−) Ctrl (GraphPad Prism^®^ version 7.0, two-way ANOVA, Tukey test were used for statistical evaluation. Statistical significance level was accepted as *p* < 0.05)

Studies claimed that germanium functionalized graphene could be integrated into biomedical devices because of its antimicrobial and hemocompatibility properties. If a graphene material is combined with germanium, there would be a greater antimicrobial and biocompatible property compared to the bare graphene molecule [49]. Furthermore, positively charged Ge NPs were shown to have a cytotoxic effect on the human colonic adenocarcinoma Caco-2 and rat alveolar macrophage NR8383 cells but, dextran, carboxylic acid, and PEG coating coupled with Ge NPs displayed no toxicity on the cell lines. These analyses indicated that surface chemistry was the main player in the biocompatibility of a material [50, 51]. In addition to these studies, in vitro toxicity analysis of corroding tungsten coils on human pulmonary arterial endothelial, human dermal fibroblasts, and smooth muscle cells showed that the degradation rate of tungsten coils was very slow and degraded tungsten particles had no toxicity on the cell lines. Local cytopathologic effects only seemed when particle concentration increased to very high rates (>50 mg/ml) [52]. Besides, diamond-like carbon with tungsten films was produced by using magnetron sputtering coating and this coating was claimed to have higher chemical resistance, lower roughness, and higher biocompatibility in contrast to common DLC films [53]. Supporting these studies, our investigations exhibited that W–Ge coatings had greater cellular spreading, attachment, and biocompatibility properties compared to each metal’s unique feature. Fluorescent microscopy images of cell morphology were investigated using Hoescht 33258 nuclei staining. According to the results, HDFa cell cultures were well spread, and the attachments were seemed favorable after 24 h of seeding on the germanium–tungsten coated borosilicate surface. The number of viable cells was found similar to the negative control (polystyrene cell culture plate bottom surface), and cell nucleus were investigated as well round shapes and no visible nuclear mutations. On the other hand, HDFa cells were not seemed to spread or attached in a high ratio on the uncoated borosilicate surface. Also, degraded and distorted nuclei bodies were analyzed to be excessive on the uncoated surfaces (Fig. 8). Also, the cell viability rate on W–Ge coated borosilicate glass (93%) was found to be very high compared to uncoated glass samples (21%) and there was no significant difference between Ge–W coatings and polystyrene cell culture plates in the aspect of the attached cell numbers. Moreover, the MTT cell viability assay puts forth that there was no significant difference between the HDFa cell number on the W–Ge coated surfaces and Polystyrene cell culture plate bottom surface. On the contrary, cell numbers on the uncoated borosilicate surface were significantly decreased and cell viability was found to be reduced to about 21% (Fig. 9).

**Fig. 10:**
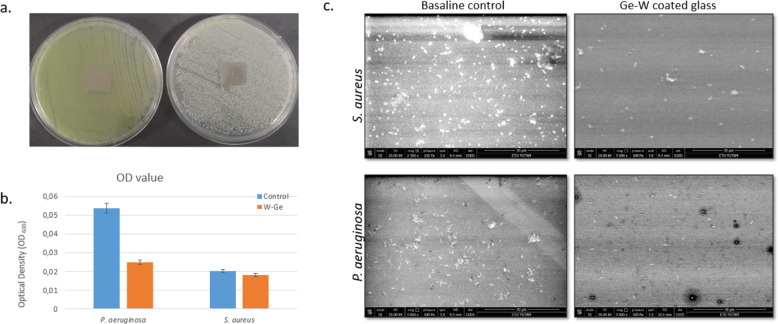
SEM morphologies of the *S. aureus* and *P. aeruginosa* biofilm on borosilicate glasses (baseline control) and W–Ge coated surface (magnification: ×5000)

In the holo-inhibition test used to measure antimicrobial activity, the area without reproduction was evaluated. However, no inhibition zone was observed in either the baseline control group or samples (Fig. 10a). It was found that Ge–W coating samples did not show antimicrobial activity against *S. aureus* and *P. aeruginosa*. This result shows that the coating material is not released into the petri dish. The media may not have been suitable for W–Ge release [8]. However, when we repeat the experiment with a nutrient agar medium, no inhibition zone was observed [8]. OD values of *P. aeruginosa* and *S. aureus* solution obtained from borosilicate glasses coated with tungsten–germanium with trypsin treatment were 0.025 and 0.018 nm, respectively. In contrast, the OD values of baseline for *P. aeruginosa* were 0.0534 and for *S. aureus* 0.020 nm as shown in Fig. 10b. The adherence of the bacteria was conducted for W–Ge coated and uncoated surface sample using SEM images (Fig. 10c). This indicates the antibiofilm properties of W–Ge coated sample against both *S. aureus* and *P. aeruginosa*. In the current study, glass without coating is used as a baseline control [54]. It is clearly seen that the level of adherence in bacteria decreases in W–Ge coated samples. Therefore, it is understood from SEM images that it shows antibiofilm activity.

## Conclusions

W–Ge composition films were deposited using the reactive magnetron sputtering technique first time in the literature. The WGe_2_ chemical compound was observed with a dense structure in (110) crystalline phase. The mean fraction coefficient of the W–Ge film on Ti buffer layer was obtained ~0.3, which was quite lower than the uncoated 316 L substrate. The adhesion strength value of the coated film was obtained 32.8 N. The static corrosion analysis showed that there was no significant change in surface morphology and the atomic ratio of W–Ge coatings after 7 days of DMEM immersion. Also, biocompatibility analysis showed that HDFa cell cultures were favorably attached to the sample surfaces, and cells were spread with favorable homogeneity. Cell viability assay puts forth that there was no significant difference between W–Ge coated surfaces and the cell culture plate. Finally, the antibiofilm activity of coatings was shown by using two different pathogenic bacteria strains (*S. aureus* and *P. aeruginosa*).
